# Genomic insights into *Vibrio cholerae* O1 responsible for cholera epidemics in Tanzania between 1993 and 2017

**DOI:** 10.1371/journal.pntd.0007934

**Published:** 2019-12-23

**Authors:** Yaovi Mahuton Gildas Hounmanou, Pimlapas Leekitcharoenphon, Egle Kudirkiene, Robinson H. Mdegela, Rene S. Hendriksen, John Elmerdahl Olsen, Anders Dalsgaard

**Affiliations:** 1 Department of Veterinary and Animal Sciences, University of Copenhagen, Copenhagen, Denmark; 2 National Food Institute, Technical University of Denmark, Lyngby, Denmark; 3 Department of Veterinary Medicine and Public Health, Sokoine University of Agriculture, Morogoro, Tanzania; 4 School of Chemical and Biomedical Engineering, Nanyang Technological University, Singapore city, Singapore; Institut Pasteur, FRANCE

## Abstract

**Background:**

Tanzania is one of seven countries with the highest disease burden caused by cholera in Africa. We studied the evolution of *Vibrio cholerae* O1 isolated in Tanzania during the past three decades.

**Methodology/Principal findings:**

Genome-wide analysis was performed to characterize *V*. *cholerae* O1 responsible for the Tanzanian 2015–2017 outbreak along with strains causing outbreaks in the country for the past three decades. The genomes were further analyzed in a global context of 590 strains of the seventh cholera pandemic (7PET), as well as environmental isolates from Lake Victoria. All Tanzanian cholera outbreaks were caused by the 7PET lineage. The T5 sub-lineage (*ctx*B3) dominated outbreaks until 1997, followed by the T10 atypical El Tor (*ctx*B1) up to 2015, which were replaced by the T13 atypical El Tor of the current third wave (*ctx*B7) causing most cholera outbreaks until 2017 with T13 being phylogenetically related to strains from East African countries, Yemen and Lake Victoria. The strains were less drug resistant with approximate 10-kb deletions found in the SXT element, which encodes resistance to sulfamethoxazole and trimethoprim. Nucleotide deletions were observed in the CTX prophage of some strains, which warrants further virulence studies. Outbreak strains share 90% of core genes with *V*. *cholerae* O1 from Lake Victoria with as low as three SNPs difference and a significantly similar accessory genome, composed of genomic islands namely the CTX prophage, Vibrio Pathogenicity Islands; toxin co-regulated pilus biosynthesis proteins and the SXT-ICE element.

**Conclusion/Significance:**

Characterization of *V*. *cholerae* O1 from Tanzania reveals genetic diversity of the 7PET lineage composed of T5, T10 and T13 sub-lineages with introductions of new sequence types from neighboring countries. The presence of these sub-lineages in environmental isolates suggests that the African Great Lakes may serve as aquatic reservoirs for survival of *V*. *cholerae* O1 favoring continuous human exposure.

## Author summary

The seventh cholera pandemic has claimed >250,000 reported cases and 13,078 deaths until 2018 in Tanzania. To understand the epidemiology and to guide control, we used genomics to study *V*. *cholerae* O1 isolated in Tanzania during the past three decades. Tanzanian cholera outbreaks were caused by the T5, T10 and T13 sub-lineages of the 7PET lineage of *V*. *cholerae* O1 with some strains showing an unusual 100-bp deletion on the CTX prophage. From 1993 to 2017, most sub-lineages found in patients were also found in the aquatic environment and the close phylogenetic relationships between strains from the two niches suggest that the African Great Lakes may act as a reservoir for cholera outbreak strains. Moreover, we reported clonal transmission at regional and global scale favored by population displacements. Regional collaborative efforts are advised for effective cholera control.

## Introduction

In 1974, cholera reached Tanzania on the shores of Lake Nyasa bordering Malawi [1], and has since caused recurrent outbreaks of varying magnitudes almost every year resulting in over 250,000 reported cases and 13,078 deaths until 2018 [2,3]. In Africa, the different epidemics could all be traced back to a single lineage from South Asia, which has been introduced at least 11 times since the first epidemic in the 1970s [4]. The ongoing seventh cholera pandemic is characterized by multiple waves of *V*. *cholerae* O1 strains associated with various genotypic markers mainly variations in the *ctx*B gene on the CTX prophage [4,5]. To understand the evolution of *V*. *cholerae* O1 requires genome-wide analyses at national and regional scales [6].

Previous analysis of *V*. *cholerae* O1 from the 2015 cholera outbreak in Tanzania revealed that strains involved in initial outbreaks around refugee camps formed two distinct genetic lineages both different from other strains associated with the countrywide outbreak occurring later in the same year [7]. This indicates the occurrence of heterogeneous *V*. *cholerae* O1 through introductions of different sub-lineages into the country at different time points. Studies have also indicated aquatic environments as a potential source for cholera outbreak strains in Tanzania [8,9].

Here, we analyze 22 *V*. *cholerae* O1 from the 2015–2017 cholera outbreak in Tanzania in a national and global context along with strains recovered from Lake Victoria aiming to investigate their evolution, including determinants of pathogenicity and antimicrobial resistance. Lessons learnt from these past outbreak strains provide evidence of cross-border spread of *V*. *cholerae* O1 in the East African region and call for integrated collaborations of the different concerned health authorities to proactively establish joint control strategies to circumvent future cholera epidemics in the region.

## Material and methods

### Study area and strains collection

**Fig 1:**
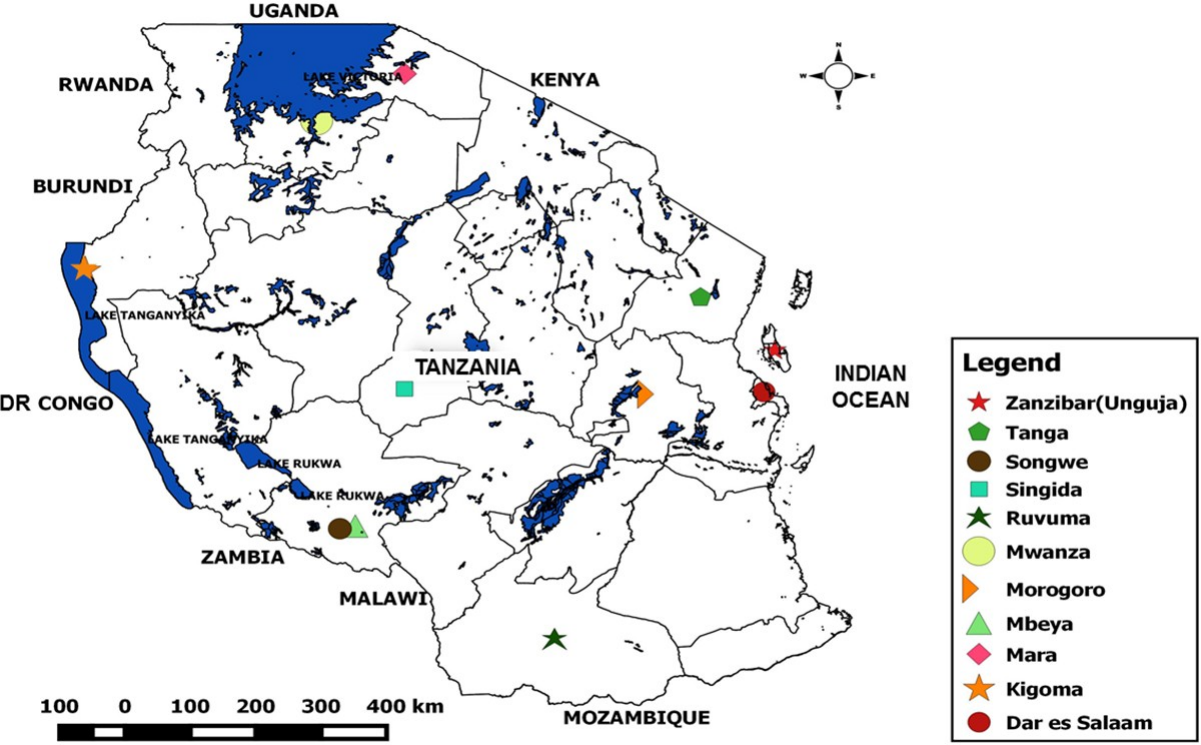
Sampling area. The *V*. *cholerae* O1 strains analyzed originated from regions listed in the legend box of the map. Map constructed with QGIS version 2.12.3 (https://www.qgis.org) using the GPS coordinates recorded from our sampling sites and Tanzanian country shape files obtained from DIVA-GIS (http://www.diva-gis.org/gdata).

The United Republic of Tanzania is an East African country and part of the African Great Lakes Region [10]. We studied clinical *V*. *cholerae* O1 strains and publicly available genomes of *V*. *cholerae* O1 from eleven regions of mainland Tanzania and Zanzibar originating between 1993 and 2017 (Fig 1, S1 Table). *V*. *cholerae* O1 isolated between 2015 and 2017 from cholera patients in Ruvuma, Songwe, Dar es Salaam, Morogoro, Mwanza, Mbeya, Kigoma and Tanga were obtained from the National Health Laboratory Quality Assurance and Training Centre of the Ministry of Health in Dar es Salaam (Fig 1). *V*. *cholerae* O1 isolated during the 2016–2017 cholera outbreak from Zanzibar were obtained from Mnazi Mmoja Hospital of the Ministry of Health and Social Affairs. Overall, two strains per region from mainland Tanzania and six strains from Zanzibar resulting in 22 strains in total were confirmed as *V*. *cholerae* O1 and subjected to antimicrobial susceptibility testing as previously described [9], and whole genome sequencing (WGS). Public genomes of clinical *V*. *cholerae* O1 isolated between 1993 and 2015 (n = 23) [4,7] and recent environmental *V*. *cholerae* strains from Lake Victoria, Tanzania (n = 9) [9] were obtained from the Genbank and the European Nucleotide Archives (ENA) and included in the phylogenetic analyses (S1 Table).

### DNA extraction, whole genome sequencing and genome assembly

DNA from the 22 *V*. *cholerae* O1 isolates was extracted using the automated Maxwell DNA extraction machine (Promega Maxwell RSC, Wisconsin, USA) and sequencing was performed on a Miseq (Illumina, Inc., San Diego, CA, USA) as previously described [9] at the University of Copenhagen, Denmark. Raw sequences were submitted to ENA (Accession number PRJEB30604). Reads were assembled using SPAdes v. 3.9 [11] and assemblies were annotated using Prokka (v. 1.12-beta) with default settings, using barrnap 0.7 for rRNA prediction [12].

### Characterization of *V*. *cholerae* O1 from Tanzania

Sequenced strains were analyzed using the online tools from the CGE platform (https://cge.cbs.dtu.dk/services/cge/) with default settings as previously described [9]. This included identification of *V*. *cholerae* serogroup-specific genes (*rfbV*-O1, *wbfZ*-O139), biotype-specific genes (*ctx*B, *rst*R, *tcp*A), major virulence genes, and VC2346 specific for the seventh cholera pandemic [13,14]. Detection of genomic islands of *V*. *cholerae* VPI-1, VPI-2, VSP-1, VSP-2 and the Type VI secretion system (T6SS) proteins was carried out using MyDbFinder 1.2. Furthermore, MyDbFinder 1.2 [14] coupled with nucleotides BLAST served for genotyping of the strains based on the *ctx*B of the CTX prophage that they carried. The *ctx*B of *V*. *cholerae* N16961 (AE003852) served as reference for *ctx*B3 to search for prototype El Tor strains. The *ctx*B1 of *V*. *cholerae* O395 (CP001235) was used to identify altered El Tor strains of the early third wave of the seventh pandemic, whereas the point mutation (C to A) at position 58 in the *ctx*B1 making it *ctx*B7 [15] served to identify strains of genotype *ctx*B7 of to the current third wave of the seventh pandemic. ResFinder 3.1 [16] with default options assessed acquired antimicrobial resistance (AMR) genes. MyDbFinder 1.2 [17] was used to detect the SXT integrative conjugative element, class 1 integrons, and the presence of mutations in the DNA gyrase (*gyr*A gene) and in the DNA topoisomerase IV (*par*C gene) [14]. Search for plasmids was conducted using PlasmidFinder 1.3, MyDbFinder 1.2 tools, with cryptic plasmid replicons [9] and Blast atlas using GView (https://server.gview.ca/) to assess occurrence of plasmid replicons in the sequences. *In-silico* MLST was performed [13] based on internal fragments of the seven housekeeping genes: *adk*, *gyrB*,*metE*, *mdh*, *pntA*, *purM*, and *pyrC* using MLST 2.0 [17]. The included public available genomes have previously been reported [4,7,9] and were included for comparative analysis. The sequence types of the already published genomes were originally not reported [4,7], but we determined these using MLST 2.0 [17]. We localized resistance genes on plasmids from the public available genomes containing the IncA/C2 plasmid [4] using Blast Atlas in GView (https://server.gview.ca/). Likewise, we analyzed clinical *V*. *cholerae* O1 from previous studies [4,7] for deletions on the CTX prophage and the SXT conjugative elements by mapping the reads against the reference *V*. *cholerae* 2010EL-1786. We searched antimicrobial resistance genes and did *ctx*B genotyping and analysis of all major virulence genes as described above in the clinical *V*. *cholerae* O1 strains reported by Kachwamba et al [7], as they did not report such characteristics. The environmental strains were characterized and reported in a previous study [9] but were used in the present study for pangenomic comparison with the 2015–2017 outbreak strains for in-depth genomic analyses and for the overall phylogenetic evolution of Tanzanian *V*. *cholerae* since 1993 through 2017.

### Phylogenetic and pan-genome analyses

The phylogenetic relationship between *V*. *cholerae* O1 that caused different outbreaks in Tanzania from 1993 to 2017 was assessed along with strains recovered from the environment using raw reads and trimmed assemblies in CSIPhylogeny version 1.4 with default options for a local single nucleotide polymorphism (SNP) analysis [18]. All Tanzanian strains were then placed in a global phylogenetic context of 590 genomes of the seventh cholera pandemic to identify the global genetic relatedness and diversity of the Tanzanian strains. The pre-seventh pandemic *V*. *cholerae* O1 strain M66-2 was used to root the trees. The newick files obtained in CSIPhilogeny 1.4 were annotated and visualized in iTOL [19].

We conducted a pangenome analysis for a genome-wide comparison between selected *V*. *cholerae* strains obtained from Lake Victoria (n = 9), Tanzania [9] and the clinical strains that caused cholera in 2015 to 2017 (n = 22). Annotated .gff files were used as an input to Roary (v. 3.7.0) pangenome analysis tool [20]. The binary presence/absence data of accessory genes produced in Roary was used to calculate the associations between all genes in the accessory genome and the selected traits of the isolates by employing the Scoary (v. 1.6.11) tool [21]. The accessory genome tree was visualized in phandango [22].

## Results and discussion

### Genomic characteristics, local phylogeny and pan-genome analysis of Tanzanian *V*. *cholerae* O1

**Fig 2:**
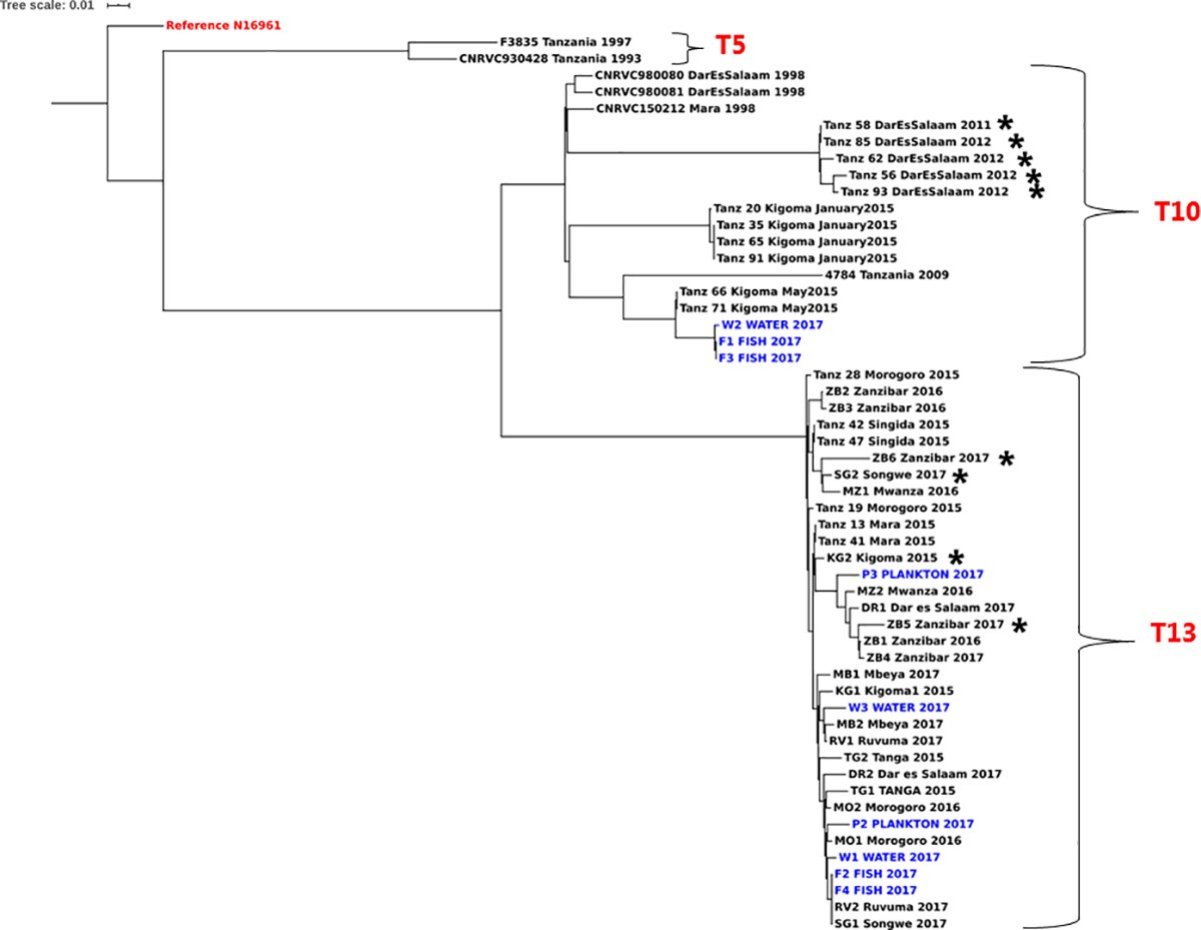
Maximum likelihood tree of *V*. *cholerae* O1 isolated in Tanzania from 1993 to 2017 along with strains from Lake Victoria (in blue). The reference strain *V*. *cholerae* N16961 was used to root the tree. Strains with the 100bp deletion in *ctx*A are marked with a star (*) and the T sub-lineages [4] of each phylogenetic cluster are indicated in brackets.

*V*. *cholerae* associated with cholera in Tanzania, belong to serogroup O1, as they possess the *rfvB-*O1 gene (Table 1, S1 Table). All Tanzanian strains, including isolates from Lake Victoria are of the seventh pandemic lineage 7PET, possessed the seventh pandemic-specific gene (VC2346) and differ from the reference seventh pandemic El Tor strain N16961 with a maximum of 160 SNPs (S2 Table, sheet 1). In agreement with previous reports, we confirmed that strains from 1993 through 1997 were all the prototype El Tor biotype (*ctxB*3) *V*. *cholerae* of sub-lineage T5 [4]. Cholera outbreaks occurring from 1998 until 2017 were caused by strains of the atypical El Tor biotype carrying either *ctx*B1 or *ctx*B7, while having *rst*R and *tcp*A of the typical El Tor biotype (Fig 2, Table 1). This coincides with the period of emergence of the hybrid biotype conferred by *ctx*B1 genes and associated with cholera outbreaks, which has since replaced the typical El Tor biotype in recent outbreaks [4,5,23]. These hybrid strains are known for their ability to produce more cholera toxin than the prototype El Tor biotype strains causing a more severe diarrhea [24]. The 1993 and 1997 strains belong to the first wave of the seventh cholera pandemic and the T5 sub-lineage of 7PET. Clinical strains from 1998 up to 2012 and strains F1, F3 and W2 isolated from Lake Victoria in 2017 belong to the early part of wave III (*ctx*B1) of the seventh pandemic and part of the African T10 cluster, confirming previous reports [4]. The Kigoma refugee camos outbreak of May 2015 [25] also belong to this cluster.

**Table 1:** Genome characteristics of *V*. *cholerae* O1 isolated in Tanzania from 1993 to 2017.

Isolation year	Biotype	MLST	*ctx*B (Wave)	Resistance (SXT/R391)	T sub-linages	References
1993–1997	Prototype El Tor	ST69	*ctx*B3 (wave 1)	ICEVchHai1_*del (floR,strA/B,sul2) + 400bp gap in floR	T5	[4]
1998–2009	Atypical El Tor	ST69	*ctx*B1 (early wave 3)	ICEVchHai1	T10	[4,5]
2011–2012	Atypical El Tor	ST69	*ctx*B1 (early wave3)	ICEVchHai1_del(floR,strA/B,sul2) + 400bp gap in floR	T10	[7]
2015 (Kigoma January)	Atypical El Tor	ST515	*ctx*B1 (early wave 3)	ICEVchHai1	T10	[7]
2015–2017	Atypical El Tor	ST69	*ctx*B7 (current wave 3)	ICEVchHai1_del(floR,strA/B,sul2)	T13	This study
2017 (P2, P3, F2, F4, W1, W3)	Atypical El Tor	ST69	*ctx*B7 (current wave 3)	ICEVchHai1_del(floR,strA/B,sul2)	T13	[9]
2017 (F1, F3, W2) + Kigoma May 2015	Atypical El Tor	ST69	*ctx*B1 (early wave 3)	ICEVchHai1_del(floR,strA/B,sul2)	T10	[7,9]

*del: deletions in specified genes.

**Fig 3:**
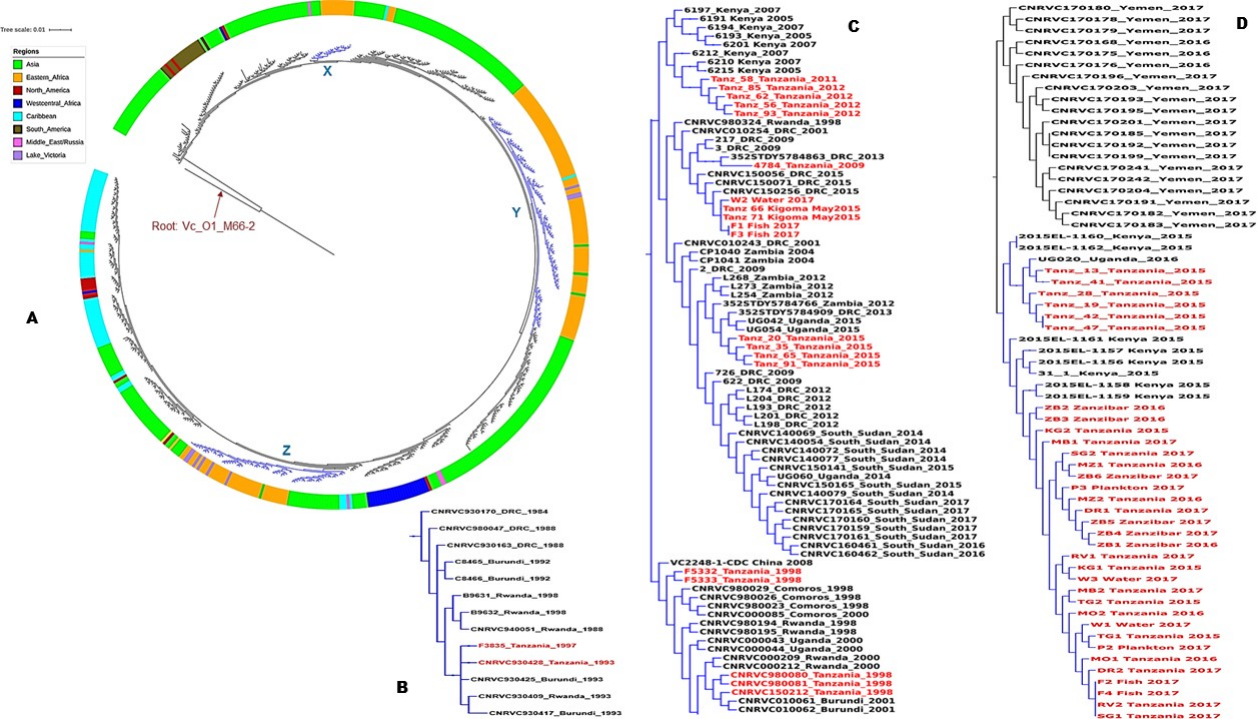
SNP-tree showing global phylogenetic relationships of *V*. *cholerae* O1 genomes by regions. The blue clades labeled X, Y and Z in panel A indicate Tanzanian strains within the T5, T10 and T13 transmission events, respectively. Panel B is a zoom into the clade X showing the Tanzanian T5 strains. Panel C is the Y clade of Tanzanian strains within a T10 cluster. Panel D displays the clade Z indicating T13 strains including Tanzanian strains. In panels B, C and D, the Tanzanian clinical and environmental strains are highlighted in red.

*V*. *cholerae* O1 strains from 2013 were not available and there was no cholera reported in Tanzania in 2014 [2]. Strains isolated in outbreaks occurring after 2014 except for the those responsible for the Kigoma outbreaks in Janurary and May 2015, contain *ctx*B7 of the current third wave within the seventh cholera pandemic and belong to the T13 sub-lineage. Compared to T5 and T10 strains that occurred in the previous years, this shows significant genomic diversity of *V*. *cholerae* responsible for outbreaks in Tanzania overtime in line with the variation previously reported across the continent [4,5]. T13 strains are responsible for the ongoing cholera outbreak in Eastern Africa and Yemen [26,27]. Strains of the T13 sub-lineage formed a separate cluster on the local phylogenetic tree (Fig 2) and seem to occur in Tanzania after 2014, a time that corresponds with the global emergence of this sub-lineage [26]. Our clinical samples isolated between 2015 and 2017 are most closely related to *V*. *cholerae* O1 isolated in Lake Victoria in 2017 with as low as three SNPs difference and the environmental isolates also containing *ctx*B7 and being part of the T13 sub-lineage (Fig 2). This confirms our previous findings [9] and suggests a connection between environmental and outbreak strains where the isolates from the Lake could be either outbreak strains released into the environment through fecal contamination, e.g. sewage or they could be the source of the outbreak suggesting an environmental reservoir of *V*. *cholerae* O1 as described in Thailand, Cameroon and previously in Tanzania [8,13,14]. Isolates F1, F3 and W2 isolated in 2017 from Lake Victoria were revealed to belong to the sub-lineages T10 and are genetically related to pandemic strains circulating in the country since 1998 until 2015. This suggests an environmental survival of the strains even when outbreaks have ceased in people, favoring resurgence of epidemics overtime, with Lake Victoria serving as a reservoir as is also the case for Lake Chad [9,13]. Of the twenty-two 2015–2017 strains sequenced in this study, none was T10so their presence in the lake could not be directly linked to the discharge of urban sewage emanating from the ongoing outbreaks and the environment could remain a potential reservoir for resurgence of toxigenic *V*. *cholerae* O1. However, since only a few samples were sequenced in this study from the 2015–2017 outbreak, we cannot rule out the possible presence of T10 sub-lineage in the outbreak and their subsequent discharge in the lake justifying the close genetic relatedness between our environmental isolates F1, F3 and W2 and the clinical T10 strains from the country (Fig 2, Fig 3C). Moreover, despite the well-described environmental reservoirs for *V*. *cholerae* [28–30], and the evidence of different sub-lineages of the seventh pandemic strains in the aquatic environment, it remains unclear if patients or the Lake Victoria was the original source of the isolates.

Most Tanzanian *V*. *cholerae* O1 strains isolated after 2014 are T13 and belong to the common MLST type ST69 [14]. Nevertheless, a group of T10 strains caused an outbreak in the city of Kigoma in January 2015 [7] belonging to ST515; a type that had not occurred before in Tanzania and which formed a separate cluster in the phylogenetic tree (Fig 2) within the T10 cluster. These strains belonged to a separate genotype when previously compared by MLVA typing with other genomes from late 2015 [7]. We found that the T10 strains of ST515 most likely originated from the neighboring Democratic Republic of Congo (DRC), a country known for recurrent cholera outbreaks [31] and other neighboring countries where they have caused outbreaks between 2012 and 2013 (Panel C, Fig 3).

Although T10 strains have been occurring in Tanzania since 1998 through 2015, the sequence type ST515 of the Kigoma strains was different and can be distinguished from the circulating Tanzanian T10 (ST69) (Panel C, Fig 3). However, in May 2015 the refugee camp outbreak in Kigoma caused by the locally circulating T10 ST69 strains only occurred around the refugee camp and could be attributed to the regional spread of this genotype likely favored by population displacement and refugees that fled in at the time due to conflicts in Burundi [7,25]. ST515 has been circulating in DRC before its occurrence in Kigoma in January 2015, thus, the presence of refugee camp in the area and the interaction between local fishermen and refugees from DRC and Burundi could have favored the introduction of T10 ST515 into Tanzania since this type occurred only around Kigoma near the DRC border. T10 were not related to the T13 *V*. *cholerae* O1 strains (at least 108 SNPs apart) involved in the countrywide cholera outbreaks later in the same year [7] (Fig 2). It was not possible to identify any genome sequences of *V*. *cholerae* associated with outbreaks in Burundi between 2010 and 2015, a period where most refugees fled into Tanzania. The observed regional transmission is consistent with cholera outbreaks in Tanzania being caused by diverse strains even within the same year and underlines that regional collaborative efforts are required for effective cholera control in countries located around the African Great Lakes.

The occurrence of virulence-associated genes and pathogenicity islands among the *V*. *cholerae* O1 sequenced in this study was similar to that of strains from previous studies [4,7,9] (S1 Table). Major virulence-associated genes such as *ctxA*, *ctxB*, *zot*, *ace*, *tcpA*, *hlyA*, *mshA*, *rtxA*, *ompU*, and *toxR*, as well as *Vgr*G, *Vas*, *Tsi* proteins of the type VI secretion system, glucose metabolism genes, *als* and the flagella-mediated cytotoxin gene *mak*A were present in all sequenced strains. Moreover, our sequences contained Vibrio Pathogenicity Islands mainly VPI-1 and VPI-2 as well as VSP-1 and VSP-2 normally found in strains of the seventh pandemic.

Nevertheless, a 100-bp nucleotide deletion was observed in the cholera enterotoxin gene (*ctx*A) between positions 1042170 and 1042270 in strains Kg2, Sg2, Zb5 and Zb6 isolated between 2015 and 2017 (S1 Fig) as well as in the published genomes of *V*. *cholerae* O1 isolated in 2011 and 2012 [7]. To confirm this, we repeated DNA extraction from fresh cultures of the four mentioned outbreak strains and re-sequenced them with results remaining the same. The 100-bp deletion was also confirmed by mapping the reads to the reference *V*. *cholerae* 2010EL-1786. The concerned strains were negative for *ctx*A in PCR, although the strains originated from stool samples of cholera patients. It remains to be shown how this deletion affects cholera enterotoxin production. These deletions are however, not monophyletic because they are found in strains belonging to two separate clusters from T10 and T13 (Fig 2) suggesting that they could be involved in recombination events since the deletions are occurring within known mobile elements and such events have been reported to affect the structure of *V*. *cholerae* populations [32]. Differences in the clinical relevance of these recombined strains compared to other strains can however not be demonstrated with the current data. Studies in Mozambique [33] and Mexico [34] have reported outbreak strains of *V*. *cholerae* O1 lacking *ctx*A. Moreover in Bangladesh, *V*. *cholerae* isolated from a cholera patient lacked the entire CTX bacteriophage encoding *ctxAB* genes where toxigenic *ctx*A-positive strains co-infected the same individual at the same time [35,36]. The phylogenetic difference between the two strains in that patient suggests that different populations of *V*. *cholerae* can occur in the same patient at a given time.

**Fig 4:**
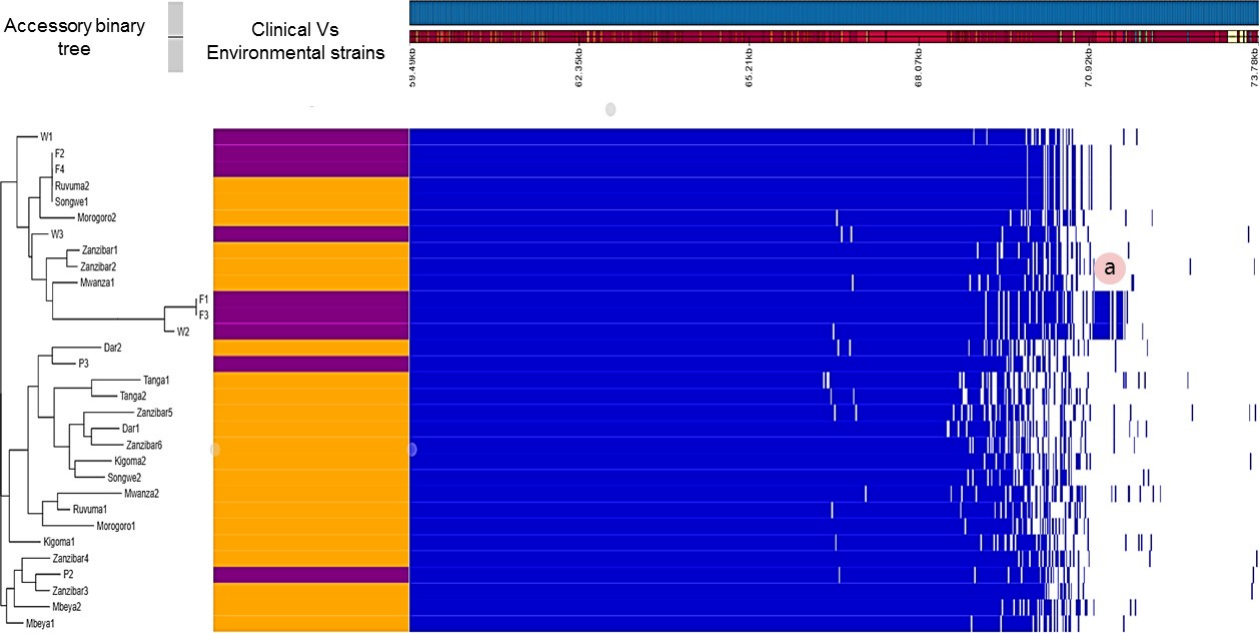
Accessory genome content of pandemic *V*. *cholerae* from Tanzania (2015–2017 in orange) versus *V*. *cholerae* O1 isolated in Lake Victoria (purple). The tree at the left shows the accessory binary tree of the accessory genome indicating that clinical strains F2 and F4 are identical to environment strains Rv2 and Sg1. The blue boxes mark presence of genes and white gaps represent absence of gene products. The label (a) shows strains W2, F1 and F3 containing a unique region of proteins from the VSP-2 genomic island like the murin DD-endopeptidase *Mep*M that are absent in other strains.

When *V*. *cholerae* O1 strains isolated from Lake Victoria [9] were compared to the latest outbreak strains using a genome-wide approach, we observed that the clinical and environmental isolates share a core genome of 3,321 genes, being the number of genes common to all 31 analyzed strains, out of a total pan-genome size of 3,687 (90.07%) (Fig 4). As shown in the core genome phylogeny where clinical and strains from the Lake were highly related with as low as 3 SNPs apart, the accessory genome also shows that two fish isolates (F2 and F4) are identical to two isolates from patients (Fig 4), confirming the connection between isolates from the environment and from patients. This finding supports our initial argument of an environmental reservoir for *V*. *cholerae* as a potential source of outbreaks [9] and persistence of pandemic strains in the environment confirming why *V*. *cholera* O1 has persisted across the three major niche dimensions namely space, time, and habitat [37]. Nevertheless, we still cannot be conclusive on the direction of contamination between the environment and patients. The core-genome is made amongst others of the outer membrane protein genes, the kinase two-component signal transduction histidine-proteins, the chemotaxis proteins and corroborate previous findings that define species-specific genes of *V*. *cholerae* supporting environmental adaptation [38]. The accessory genome of the analyzed genomes is however, organized in two main clusters of 110 genes (Fig 4). Between clinical strains and those recovered from the environment, no gene from the accessory genome showed a significant predilection to either of the niches (Benjamini p-value >0.05), substantiating a strong genetic relatedness even at accessory genome level between clinical and environmental *V*. *cholerae* O1 in Tanzania. This finding is however contrary to previous studies that reported a clear difference between clinical and environmental *V*. *cholerae* O1 primarily due to lack of virulence-associated genes in most environmental strains [38].

The accessory genome of the analyzed strains essentially constitutes of genomic islands mainly the Vibrio Pathogenicity Islands, toxin co-regulated pilus biosynthesis proteins, the CTX prophage, and resistance genes on the SXT integrative conjugative element (Fig 4). These findings corroborates previous finding [37,39] and confirms that the CTX prophage is not part of the core genome of *V*. *cholerae* O1. The accessory binary trees (Fig 4) shows a distinct cluster of four non-T13 strains (W2, F1 and F3), with a significantly different accessory genome content (Benjamini p-value< 0.05). The accessory genome of strains recovered from the environment reveal that they are characterized by the presence of bicyclomycin resistance proteins encoded by genes acquired by horizontal gene transfer [39]. Strains W2, F1 and F3 harbored proteins belonging to the genomic island of VSP-2 like the murein DD-endopeptidase *Mep*M, that were absent in remaining strains (Fig 4, label a).

### Determinants of antimicrobial resistance

Our sequenced strains showed phenotypic resistance to streptomycin, amoxicillin-clavulanic acid and ampicillin as well as nalidixic acid. Resistance to nalidixic acid was confirmed by the presence of amino acid substitutions in *gyr*A (Ser83-Ile) and *par*C (Ser85Leu). Strains were, however, susceptible to several antimicrobials including gentamicin, ciprofloxacin, ceftazidime, tetracycline, cefotaxime and chloramphenicol. All *V*. *cholera*e O1 genomes contained resistance genes for chloramphenicol (*cat*B9) and trimethoprim (*dfr*A1/15) with the latter gene being part of the SXT element, but our sequenced strains were susceptible to chloramphenicol in phenotypic tests. Such discrepancy between phenotypic and genotypic profiles have been reported previously [40]. Moreover it has already been reported that the presence of catB9 is not associated with resistance [4].

In accordance with characterization of previous *V cholerae* O1 strains [4,7], our strains contained the SXT integrative conjugative element with genetic similarity to that of *V*. *cholerae* ICEVchHai1 and harbor the specific integrase genes of the class 1 integron, (*int*I gene). Blast Atlas analysis revealed that strains from 2015 to 2017 have approximately 10-kb nucleotide deletions on the SXT element especially in *flo*R (bp 99050 to 99200), *str*A/B (bp 100350 to 100600; 100800 to 100900; 101600 to 101850) and *sul*2 (bp 102300 to 102450) (S1 Fig) most likely resulting in phenotypic susceptibility to phenicols and sulphonamide. These deletions are characteristic for the T13 sub-lineage of *V*. *cholerae* O1 El Tor found in the current third wave of the seventh pandemic and have been previously reported in Cameroon [13] and Yemen [26]. These deletions in the ICE fragment may have caused the strains to be less resistant to antimicrobials as compared to the clinical T5 strains isolated in 1993 and 1997, which harbor conjugative IncA/C2 plasmids as reported elsewhere [4] with additional beta-lactam (*bla*_CARB-4_), and tetracycline (*tet*B) resistance. No strains isolated after 1998 contained conjugative plasmids. It seems that *V*. *cholerae* O1 clones of the third wave have lost the *Inc*A/C plasmids over the years [4,26,41].

### *V*. *cholerae* O1 from Tanzanian outbreaks in a global context

In the global context of the seventh pandemic, Tanzanian strains are located on three time-separated clusters (Panel A, Fig 3). The T5 prototype El Tor strains from 1993 and 1997 are located in a cluster of closely related genomes from India, Bangladesh and China isolated between the 1970´s and the 1990’s (Panel B, Fig 3). These strains have been circulating for nearly 20 years in Africa revealing decades long transmission chain between African countries [4]. Their relatedness to strains from Asia shown in our analysis (Panel B, Fig 3) reiterates the Asian origin of initial cholera outbreaks in Tanzania and in Africa [4]. The T10 strains isolated between 1998 and 2012, including the 2015 strains from Kigoma formed a regional cluster (Panel C, Fig 3), confirming spread of *V*. *cholerae* O1 between Tanzania and other Eastern African countries like Rwanda, Burundi, Kenya, Uganda, DRC, South Sudan, Comoros, and Zambia [4,7,27]. *V*. *cholerae* O1 isolated in Tanzania during the 2015–2017 outbreak clustered with strains from East Africa mainly the 2015 and 2016 outbreak strains from Kenya and Uganda with a maximum of 50 SNPs difference (Panel D, Fig 3 and S2 Table, sheet 2). The fact that these three neighboring countries that have Lake Victoria in common experienced outbreaks during the same period with genetically closely related strains, also found in the lake, underlines the need for regional collaboration for cholera control and the inclusion of environmental surveillance in control strategies. Moreover, all *V*. *cholerae* O1 strains isolated after 2014 until 2017 are closely related to *V*. *cholerae* O1 that caused the devastating 2016–2017 outbreaks in Yemen (Panel D, Fig 3) confirming previous reports on potential human-mediated transmission around the globe [26,42].

In conclusion, genomic analyses of *V*. *cholerae* O1 responsible for various outbreaks in Tanzania between 1993 and 2017 confirmed that the seventh pandemic El Tor strains caused all outbreaks. This lineage however has undergone significant genetic changes over time. The year 2015 for instance shows the diversity of strains causing various outbreaks in Tanzania because in that year the January outbreaks were caused by T10 ST515 strains, while in May the outbreak in the same city was caused by T10 ST69 and from August 2015 the Kigoma strains were T13. We have confirmed spread within the Eastern African countries notably between Tanzania, the Democratic Republic of Congo, Kenya and Uganda, Rwanda, Burundi, Zambia, South Sudan and Comoros, as well as a global spread between East African countries and Yemen for T10 and T13 strains. Tanzanian older epidemics clones of T5 sub-lineage however most likely originated from India, Bangladesh or China. These findings are consistent with human-mediated spread of cholera around the globe. We have documented potential aquatic environmental reservoir for *V*. *cholerae* O1 strains, which are closely related to epidemic clones with similar accessory-genome contents. Different sub-lineages of epidemic strains mainly T10 and T13 have been found in the lake substantiating survival, persistence from the lake and favor further human exposure. Tanzanian *V*. *cholerae* O1 strains show limited antimicrobial resistance and some present nucleotide deletions on the CTX prophage. The observed regional spread calls for well-coordinated cholera control efforts including environmental monitoring of *V*. *cholerae* O1 in the African Great Lakes regions, which is currently the main cholera hotspot on the African continent. We propose initiation of vaccination programs in countries whose neighbors declare cholera epidemics.

### Limitations of the study

In the present study only a limited number (n = 22) of *V*. *cholerae* O1 isolates collected between 2015 and 2017 have been analyzed from an outbreak that caused over 30, 000 reported cases between August 2015 and early 2018. Considering this limited sample size, it is difficult to rule out the possibility of occurrence of more recent T10 isolates collected in humans during the outbreaks around Lake Victoria justifying their clustering with our environmental F1, F3 and W2 isolates. Furthermore, the data presented in this study provided evidence of phylogenetic relatedness between clinical and environmental isolates of *V*. *cholerae* O1 in Tanzania but cannot indicate the direction of pathogen transfer and original source. Moreover, the identification of imported strains of *V*. *cholerae* through refugees and the occurrence of different sub-lineages over time in Tanzania and beyond in the Great Lakes region cannot effectively guide cholera control without parallel epidemiological studies and interventions from decision makers. The tools used in this study and the available data are not able to predict the next potential sub-lineages to emerge in future epidemics and their clinical relevance in order to proactively propose solutions. Furthermore, the current data does not allow to conclude on the epidemiological relevance of the identified *V*. *cholerae* O1 from cholera patients containing deletions on the *ctx*A gene, the main virulence factor for cholera toxin production.

## Supporting information

S1 Fig Nucleotide deletions in *ctx*A and on the SXT fragment of *V*. *cholerae* O1 genomes from Tanzania sequenced in this study. Observed gaps represent the areas of missing nucleotides in strains indicated in the color legend.
(TIF)

S1 Table Genomic sequence data, virulence profile and occurrence of antimicrobial resistance genes in Tanzanian *V*. *cholerae* O1 strains. (XLSX)

S2 Table Pairwise SNP differences for local and global phylogeny of 589 strains used in the global seventh pandemic tree. (XLSX)
