# Supplementary figures and images for: Genomic insights into Vibrio cholerae O1 responsible for cholera epidemics in Tanzania between 1993 and 2017
Source: PLoS Negl Trop Dis. 2019 Dec 23;13(12):e0007934. doi: 10.1371/journal.pntd.0007934 (PMC6927581; doi:10.1371/journal.pntd.0007934)

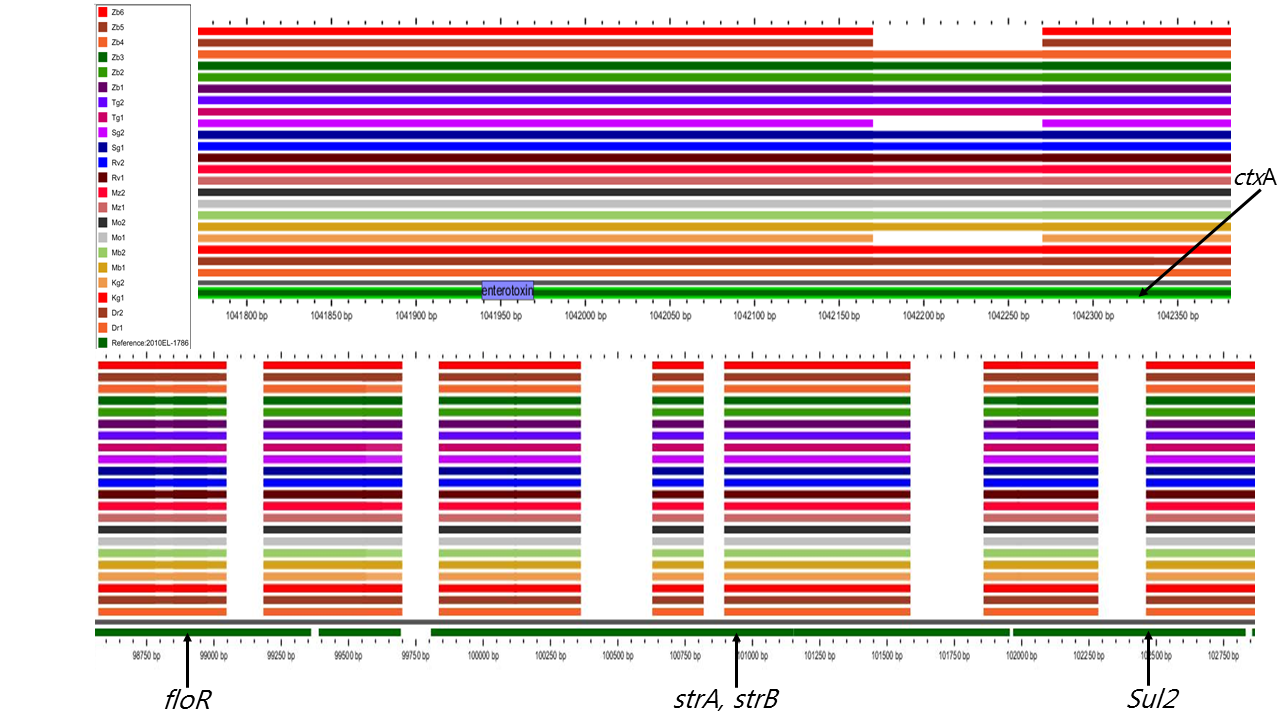

Supplement: S1 Fig — Observed gaps represent the areas of missing nucleotides in strains indicated in the color legend. (TIF) [file pntd.0007934.s001.tif]
